# Editorial: Artificial intelligence for early diagnosis of colorectal cancer

**DOI:** 10.3389/fonc.2024.1495827

**Published:** 2024-11-26

**Authors:** Luisa F. Sánchez-Peralta, Benjamin Glover, Debesh Jha, J. Blas Pagador

**Affiliations:** ^1^ Bioengineering and Health Technologies Unit, Jesus Uson Minimally Invasive Surgery Centre, Cáceres, Spain; ^2^ Imperial College London, London, United Kingdom; ^3^ Machine & Hybrid Intelligence Lab, Department of Radiology, Northwestern University, Chicago, IL, United States

**Keywords:** colorectal cancer, artificial intelligence, early diagnosis, medical imaging, polyp detection

Colorectal cancer (CRC) is generally defined as an adenocarcinoma located in the colon or rectum (1). In 2022, there were an estimated 1.9 million new CRC cases and 904,000 associated deaths worldwide, accounting for one-tenth of both new cancer cases and deaths from the disease, making CRC the third most common cancer in terms of incidence and second in terms of mortality (2). It is estimated that both incidence and mortality will increase annually, reaching one million deaths by 2045 (3).

The integration of Artificial Intelligence (AI) technologies into healthcare is revolutionising early diagnostic processes for diseases, also for CRC, enhancing both the effectiveness and efficiency of traditional diagnostic methods (4).

Fortunately, early detection of CRC increases the 5-year survival rate from 18% when detected at the most advanced stage to 88.5% when detected at an early symptomatic stage (5). Screening programs are implemented for such early detection, as tumours detected in symptomatic patients are larger and at more advanced stages than tumours detected in asymptomatic patients in a screening programme (6). Thus, the detection of CRC is critical yet challenging, often requiring operator-dependant endoscopic procedures, followed by accurate histological assessment of tissue biopsies. AI offers promising enhancements to these traditional methods, providing tools that are less invasive, more accurate, faster, and consistent.

**Figure 1:**
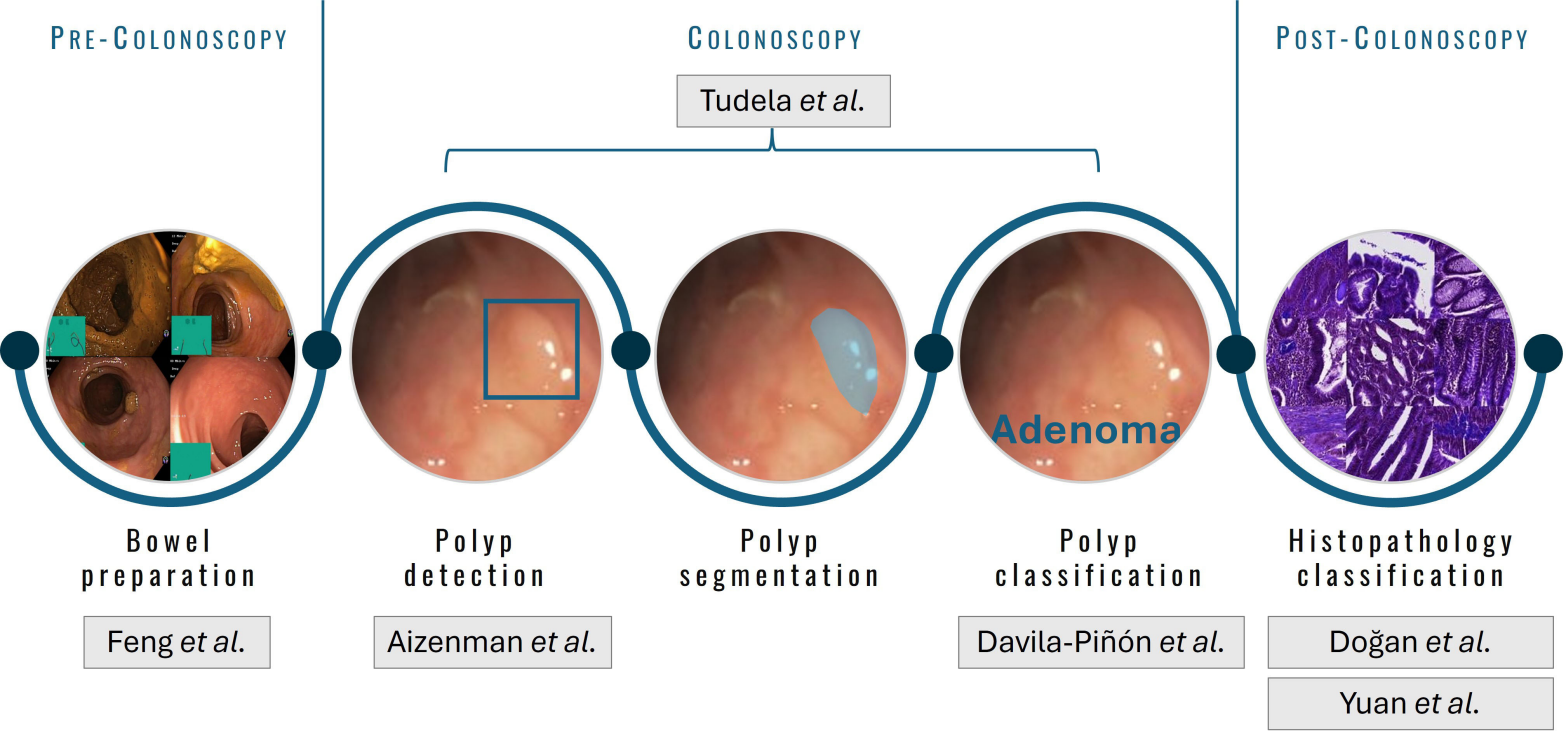
Stages in which AI can be applied in the scope of colonoscopy and colorectal cancer.

In the field of CRC, AI can be applied to different stages of the process, since evaluation of the bowel preparation, before the colonoscopy itself, to histopathology classification, once the lesion has been removed (**Figure 1**).

The included manuscripts contribute significant insights into the application of AI in early colorectal cancer diagnosis in all these steps.

Regarding bowel preparation, Feng et al. present a novel AI system, ViENDO, that evaluates bowel cleanliness in colonoscopy videos using dual Convolutional Neural Networks (CNNs). The system filters unsuitable frames and assesses bowel preparation using 3D CNN technology. ViENDO demonstrated high accuracy, suggesting its potential to automate Boston Bowel Preparation Scale (BBPS)-based assessments and enhance diagnostic efficiency.

As for polyp detection, Aizenman et al. focus on enhancing computer-aided detection (CADe) systems using deep learning. By integrating extensive datasets and simulating clinical environments, the study introduces clinically relevant metrics, significantly improving CADe systems’ utility and reducing false positives.

Davila-Piñón et al. compare the PolyDeep computer-aided detection and classification (CADe/x) system with expert endoscopists in diagnosing neoplastic lesions, including adenomas, sessile serrated lesions, and traditional serrated adenomas in narrow band imaging (NBI) images. The results reveal similar overall sensitivity and specificity between PolyDeep and endoscopists, with PolyDeep showing a higher sensitivity but lower specificity in the optical diagnosis of adenomatous polyps. While expert endoscopists still maintain better overall discriminatory ability, the CADe/x system has potential to complement and possibly enhance the detection capabilities of expert endoscopists, particularly by improving sensitivity.

With a broader scope, Tudela et al. present a comprehensive validation framework evaluates various AI methodologies for polyp detection, segmentation, and classification. This study introduces a new polyp classification dataset, CVC-HDClassif, and emphasizes on strong performance achieved in detection and segmentation. However, it also reminds us of the need for further improvement in classification accuracy especially in between adenomatous and non-adenomatous polyps. By establishing a new benchmark on three different datasets, the authors establish a new benchmark for the future research in the field of polyp characterization.

Following tissue retrieval, Yuan et al. demonstrate an AI algorithm developed through transfer learning from a polyp segmentation model. This localises CRC regions within precise grids on whole slide imaging (WSI). The method boasts high sensitivity and specificity, and addresses challenges related to AI use and applicability, by accurately labelling cancer presence in histological slides, thus demonstrating reliability and efficacy in histology applications.

Regarding to post-colonoscopy stage, Doğan et al. present a study that evaluates the role of AI in histopathological image analysis, notably using CNNs for classifying diverse tissue types within digital pathology. By comparing AI-based algorithms against manual machine learning models, the research demonstrates the superior performance of CNNs in both binary and multi-class classifications of tissues, achieving accuracies of 0.91 and 0.97, respectively. Utilizing over 100,000 images for training and 7,180 for testing, the study highlights AI’s potential to enhance diagnostic accuracy and efficiency in identifying tumour tissues and other types, marking a significant advancement over traditional methods.

These studies demonstrate how AI can significantly enhance diagnostic accuracy and efficiency in clinical settings. However, integrating AI tools into healthcare systems will further involve overcoming challenges related to ethical and societal implications, workflow integration and interoperability or interpretability (7).

Addressing integration challenges and expanding research to include diverse, multicentre trials will be crucial. This will help validate AI tools’ efficacy and safety in real-world settings, ensuring AI tools meet the diverse needs of global populations.

The emergence of AI in early CRC diagnosis signifies a significant advance toward more proactive, individualised, and minimally invasive healthcare. Continued research and development are essential to harness the full potential of AI, promising to transform CRC diagnosis and improve patient outcomes and experience.
